# Preparation and characterization of microparticles of piroxicam by spray drying and spray chilling methods

**Published:** 2010

**Authors:** M. Dixit, A.G. Kini, P.K. Kulkarni

**Affiliations:** Department of Pharmaceutics, JSS College of Pharmacy, JSS University, S.S Nagar, Mysore-570015, India

**Keywords:** Spray drying, Spray chilling, Piroxicam, Solubility, Dissolution, Crystallinity

## Abstract

Piroxicam, an anti-inflammatory drug, exhibits poor water solubility and flow properties, poor dissolution and poor wetting. Consequently, the aim of this study was to improve the dissolution of piroxicam. Microparticles containing piroxicam were produced by spray drying, using isopropyl alcohol and water in the ratio of 40:60 v/v as solvent system, and spray chilling technology by melting the drug and chilling it with a pneumatic nozzle to enhance dissolution rate. The prepared formulations were evaluated for *in vitro* dissolution and solubility. The prepared drug particles were characterized by scanning electron microscopy (SEM), differential scanning calorimeter, X-ray diffraction and Fourier transform infrared spectroscopy. Dissolution profile of the spray dried microparticles was compared with spray-chilled microparticles, pure and recrystallized samples. Spray dried microparticles and spray chilled microparticles exhibited decreased crystallinity and improved micromeritic properties. The dissolution of the spray dried microparticle and spray chilled particles were improved compared with recrystallized and pure sample of piroxicam. Consequently, it was believed that spray drying of piroxicam is a useful tool to improve dissolution but not in case of spray chilling. This may be due to the degradation of drug or variations in the resonance structure or could be due to minor distortion of bond angles. Hence, this spray drying technique can be used for formulation of tablets of piroxicam by direct compression with directly compressible tablet excipients.

## INTRODUCTION

Formulation and manufacturing of solid oral dosage forms, and tablets in particular, have undergone rapid changes and development over the last several decades. One of the most revolutionary technologies is that of direct compression. Direct compression is economical, facilitates processing without the need of moisture or heat, and involves small number of processing steps. In direct tabletting method, it is necessary to increase flowability and compressibility of the bulk powder in order to retain a steady supply of powder mixture to the tabletting machine and sufficient mechanical strength of the compacted tablets. In addition to increasing efficiency of the manufacturing process it is also important to increase bioavailability of the drug by improving the solubility of the bulk drug powder. Thus, one of the major challenges to drug development today is poor solubility, as an estimated 40% of all newly developed drugs are poorly soluble or insoluble in water(1–6). Consequently, many hydrophobic drugs show erratic and incomplete absorption from the gastrointestinal tract of animals and humans, which may lead to therapeutic failure. As a result, much research has been conducted into the methods of improving drug solubility and dissolution rates to increase the oral bioavailability of hydrophobic drugs.

Various techniques(7) such as melt adsorption(8), spherical agglomeration(9–11) and supercritical fluid processes, using different composition of solvents for preparation of microparticles, have been used to improve the dissolution rate of poorly water soluble drugs. Manipulation of the solid state by decreasing crystallinity of drug substances through formation of solid dispersion is one of the methods used for promoting drug dissolution. The solid dispersion technique(12,13) has often proved to be the most successful in improving the dissolution and bioavailability of poorly water soluble active pharmaceutical ingredients because it is a simple, economic, and advantageous technique. The concept of solid dispersion covers a wide range of systems. The enhancement in the dissolution rate is obtained by one or a combination of the following mechanisms: eutectic formation, increased surface area of the drug due to precipitation in the carrier, formation of true solid solution, improved wetability, and drug precipitation as a metastable crystalline form or a decrease in substance crystallinity. The type of solid dispersion(14) formed depends on both the carrier-drug combination and the method of manufacturing. Microwaves irradiation was used recently for the preparation of solventfree solid dispersions and for enhancement of release of the poorly soluble drugs. Spray drying(15,16) is one such technique of preparing solid dispersion and is widely used as an alternative to milling to reduce particle size. Spray chilling or spray congealing is another form of solid dispersion where the melted mass is atomized into droplets, which quickly solidify in a cool air(17–20). The advantage of spray chilling is that no additional manufacturing step is needed to pulverize the solid dispersion. In pharmacy, spray chilling has been used to prepare sustained-release formulations, to improve stability and to mask the unpleasant taste(21,22). The technique also has the advantages of being free from organic solvents compared to spray drying. The method has also been used by the food industry, for example, to encapsulate vitamins and mineral. Piroxicam (N-2-3-xylylanthranilic acid) was chosen as a poorly water soluble drug. It is one of the safest and most potent non-steroidal anti-inflammatory drugs being widely used in the market. The drug used to treat rheumatoid arthritis, osteoarthritis, and mild to moderate pain. It has low aqueous solubility and hence poor dissolution(23). The present work was conducted to improve the wet-ability, solubility and hence the dissolution of piroxicam using spray drying and spray chilling techniques.

## MATERIALS AND METHODS

### Materials

Piroxicam was obtained as a gift sample from Ipca Pharmaceutical (Mumbai, India). Isopropyl alcohol was procured from Merck (Mumbai, India). All chemicals and buffers used were of analytical grade.

### Preparation of microparticles

#### Microparticles prepared by spray drying

Spray dried particles consisted of piroxicam were prepared by dissolving the 10 g drug in the mixture of isopropyl alcohol:water (40:60 v/v). The solution was spray dried using mini spray dryer LSD -48; (Jay instrument & systems Pvt. Ltd. Mumbai) at a feed rate of 12%, a vacuum in the system at -65 mm water column, atomization pressure rate 1 kg/cm^2^, aspirator level at 35%, inlet temperature at 115 ± 2 °C and outlet temperature at 45 ± 1 °C. The formed microparticles were separated using cyclone separator, collected and stored in a desiccators at ambient temperature until ready to be used.

#### Particles prepared by spray chilling

Spray chilled particles were prepared by melting the drug (minimum 5 g) at 205 ± 5 °C. The melt was kept at 205 ± 5 °C and chilled with a pneumatic nozzle (Mini Spray Dryer LSD -48; Jay instrument & systems Pvt. Ltd. Mumbai). Air kept at 20 °C. The inner diameter of the pneumatic nozzle was 0.1 mm, the capillary length was 5 mm and the pressure was 1 Kg/cm^2^. The particles were collected, separated and stored in a desiccator.

#### Recrytallization of piroxicam

Changes in crystal lattice, being induced by solvents, can influence the physicochemical properties of the substance. Hence, the mechanical, micromeritic and dissolution properties of miroparticles were compared with commercial and recrystallized samples. Recrystallization of piroxicam was carried out using the same solvent composition as was used for spray drying piroxicam, dissolved in 40 ml of isopropyl alcohol and 60 ml of water with occasional stirring for 30 min. The crystals of piroxicam were collected by filtration and were dried at 45 °C.

### Evaluation of microparticle

#### Determination of percentage yield and drug content

The percentage yield of each formulation was determined according to the total recoverable final weight of microparticles (prepared by spray drying or spray chilling) and the total original weight of piroxicam.

Microparticles (50 mg) were triturated with 10 ml of water. Allowed to stand for 10 min with occasional swirling, and methanol was added to produce 100 ml. After suitable dilution, samples were measured at 332 nm. Drug content was determined from standard plot.

#### Differential scanning calorimetry (DSC)

A DSC study was carried out to detect possible polymorphic transition during the crystallization process. DSC measurements were performed on a DSC DuPont 9900, differential scanning calorimeter with a thermal analyzer.

#### Fourier transform infrared (FTIR) spectros-copy

The FTIR spectral measurements were taken at ambient temperature using a Shimadzu, Model 8033 (USA). Samples were dispersed in KBr powder, and the pellets were made by applying 5 ton pressure. FTIR spectra were obtained by powder diffuse reflectance on FTIR spectrophotometer.

#### X-ray analysis

X-Ray powder diffraction (XRPD) patterns were obtained at room temperature using a Philips X’ Pert MPD diffractometer, with Cu as anode material and graphite monochromator, operated at a voltage of 40 mA, 45 kV. The process parameters used were set as scan step size of 0.0170(2θ).

#### Scanning electron microscopy (SEM)

Scanning electron microscopic (Joel- LV-5600, USA, with magnification of 250x) photographs were obtained to identify and confirm spherical nature and Surface topography of the crystals.

#### Micromeritic properties

Particle size of pure sample, recrystallized sample, spray dried microparticles and spray chilled particles were determined by microscopic method using calibrated ocular micrometer. Apparent particle densities of microparticles (prepared by spray drying and spray chilling) were measured using a Pycnometer. Carr’s index was determined from powder volumes at the initial stage and after 1250 tappings to constant volume (Electolab, Mumbai). The angle of repose of microparticles (prepared by spray drying and spray chilling) and commercial crystals was measured by fixed funnel method.

#### Mechanical Property

Mechanical Property(8–10), like tensile strength of microparticles (prepared by spray drying and spray chilling) was determined by compressing 500 mg of microparticles, using hydraulic press at different ton/cm^2^ for 1 min. The compacts stored in desiccators for overnight to allow elastic recovery. The thickness and diameter were measured for each compact. The hardness of each compact was then measured using Pfizer hardness tester. The tensile strength (σ) of the compact (ton/cm^2^) was calculated using the following equation:

σ = 2F/π Dt

where, F, D and t are hardness (ton), compact diameter (cm) and thickness (cm), respectively.

### Solubility studies

The solubility(12) of piroxicam microparticles (prepared by spray drying and spray chilling) in water was determined by taking excess quantity of microparticle in 50 ml to screw-capped glass vials filled with water. The vials were shaken for 12 h on mechanical shaker. The solution was filtered through Whatmann filter paper No.1, and drug concentration was determined at 332 nm.

### Dissolution studies of microparticle

The dissolution(17) of piroxicam pure sample, microparticles (prepared by spray drying and spray chilling) and recrystallized sample was determined by using USP dissolution apparatus XXIV-Type II (Electro Lab, Mumbai). Dissolution medium was 900 ml 7.4 phosphate buffer. The amount of dissolved drug was determined using UV spectrophotometric method (UV 1601 A Shimadzu, Japan) at 332 nm.

### Determination the physical stability

To determine the physical stability of microparticles, the samples were placed in a climate chamber of 20 °C and 45% relative humidity. After 90 days, the crystallinity of piroxicam in the samples was determined by means of DSC.

## RESULTS

The solvents chosen for the spray drying were isopropyl alcohol and water. Both solvents were miscible in any proportion with each other.

The spray dried formulations were collected. The powders were free-flowing and white and in case of spray chilling yellow in color. The percentage yield of spray dried piroxicam was found to be 78%. Drug content for the spray dried formulation was found to be 98 ± 0.013. The percentage yield for spray chilled piroxicam particles was found to be 88%. Such yields are higher compared to the spray dried products. Drug content for spray chilled piroxicam particle was found to be 96 ± 0.012.

**Fig. 1:**
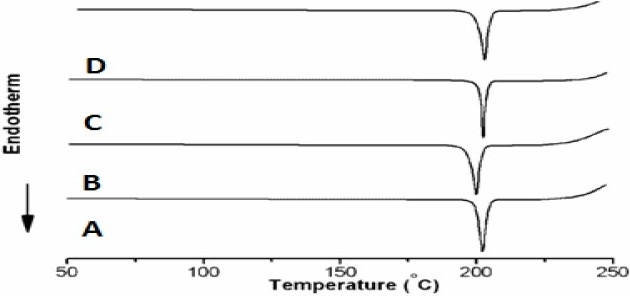
DSC of A) pure drug, B) recrystalized drug, C) chilled particles, D) spray dried particles

The DSC thermogram (Fig. 1) shows a sharp endothermic peak for all the piroxicam crystals. This one-step melt might be due to only one crystalline form (Triclinic) of the piroxicam, formed during the crystallization process, thus indicating that piroxicam did not undergo any crystal modification. The temperature range of the endothermic peak of all the piroxicam crystals lies in the range of 199.43-203.79 °C. Melting points show slight variation as the nature of the crystals might have been affected by the solvent(23).

**Fig. 2:**
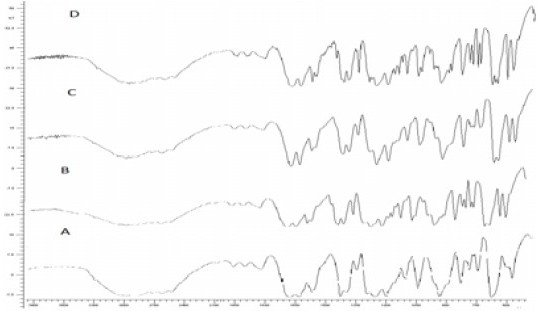
FT-IR of A-pure drug, B-recrystalized drug, Cchilled particle, D-spray dried

Infrared spectra of commercial, recrystallized, spray chilled and spray dried microparticles of piroxicam showed characteristic peaks at –NH and –OH stretching which ties at 1385 cm^-1^, 1635 or 1625 cm^-1^ (N-H-CO3 stretching vibration), 1525 cm^-1^ (secondary -NH_2_ stretching), 1440 cm^-1^ (CH_3_ AND Ar-c=c stretching), 1355 cm^-1^ (sym. – CH_3_) and 1155 and 1070 cm^-1^ or 1050-1070 cm^-1^ (-SO_2_-N-) 770 and 740 or 740 cm^-1^ (Ortho-disubstituted phenyl) (Fig. 2). The spectrum of spray chilled particles of piroxicam was slightly different from pure sample in the region of wave number between 1630 and 1780 cm^-1^(23).

**Fig. 3:**
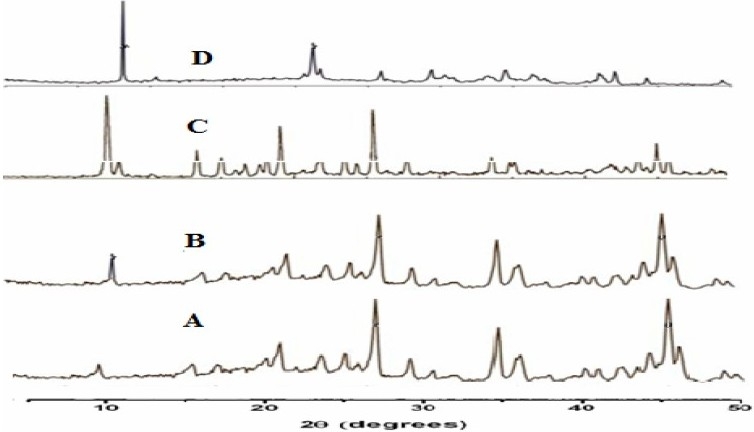
XRD of A) pure drug, B) recrystalized drug, C) chilled particles, D) spray dried particles

All the samples showed similar peak positions (2θ) in X-ray diffraction, therefore the formation of different polymorphs of piroxicam was ruled out. However, relative intensities of XRD peaks were modified (Fig. 3). This could be attributed to the markedly different crystal habits of the samples, therefore the relative abundance of the planes exposed to the X-ray source would have been altered, or may be due to differences in particle sizes(23).

**Fig. 4:**
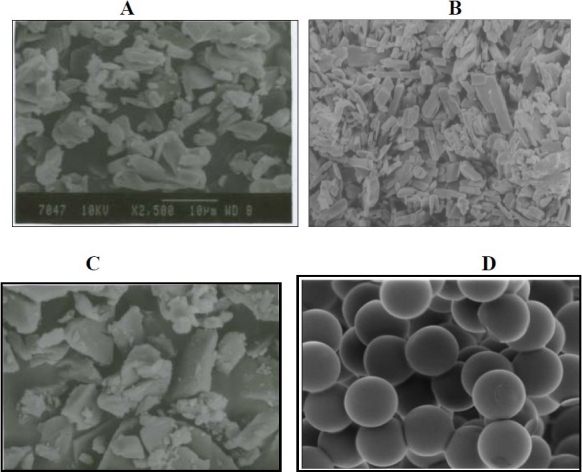
SEM of A) pure drug, B) recrystallized sample C) spray chilled particles, D) spray dried sample

The particles of pure sample were of the smallest size (4-8 μm) and they had irregular shapes. Recrystallization produced crystals with intermediate size (5-35 μm). The particles formed by spray chilling were of large size (42-70 μm) compared to the pure sample, and had irregular shape. The microparticles formed by spray drying technique had a smooth surface (Fig. 4), and were spherical in shape with small size (6-12 μm).

The Micrometrics properties of pure sample, recrystallized sample, spray chilled and spray dried microparticles of piroxicam are shown in Table 1.

**Table 1:** Micrometrics property, mechanical Property and dissolution of different samples of piroxicam.

Properties	Pure samp	Recrystallized Sample	Spray Chilled particles	Spray dried particles
Particle size (μm)	4-8	5-35	42-70	6-12
Flow rate (g/s)	No flow	No flow	1.07	2.73
Angle of repose	38.50 ± 0.022	34.13 ± 0.015	30.32 ± 0.041	27.24 ± 0.024
Tapped density (g/ml)	0.8179 ± 0.013	0.5684 ± 0.043	0.5252 ± 0.052	0.2063 ± 0.05
Bulk density (g/ml)	0.6166 ± 0.012	0.4268 ± 0.06	0.3642 ± 0.002	0.1916 ± 0.004
Carr’s index	27.17 ± 0.005	25.18 ± 0.041	23.78 ± 0.032	11.19 ± 0.036
Porosity (%)	37.10 ± 0.01	68.51 ± 0.025	69.17 ± 0.043	143.27 ± 0.018
Tensile strength (Ton/in^2^)	1.43 ± 0.026	-	2.37 ± 0.017	4.81 ± 0.011
Dissolution (% release) in 60 min	59.67 ± 0.021	71.43 ± 0.033	76.56 ± 0.018	97.85 ± 0.011

n=3, each sample was determined in triplicate.

**Fig. 5:**
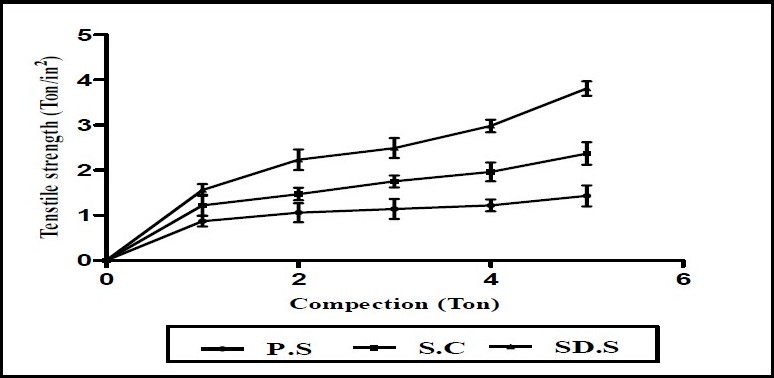
Tensile strength of pure drug (P.S), spray chilled sample (S.C), spray dried sample (SD.S). n=3, each sample was determined in triplicate.

Spray dried microparticles exhibited superior compressibility characteristics compared to pure sample and spray chilled particles (Fig. 5).

The solubility of piroxicam spray dried microparticles in water was found to be(0.0626 mg/ml) which was higher than spray chilled particles (0.0197 mg/ml), recrystallized sample (0.0094 mg/ml) and pure sample (0.0083 mg/ml)(22).

**Fig. 6:**
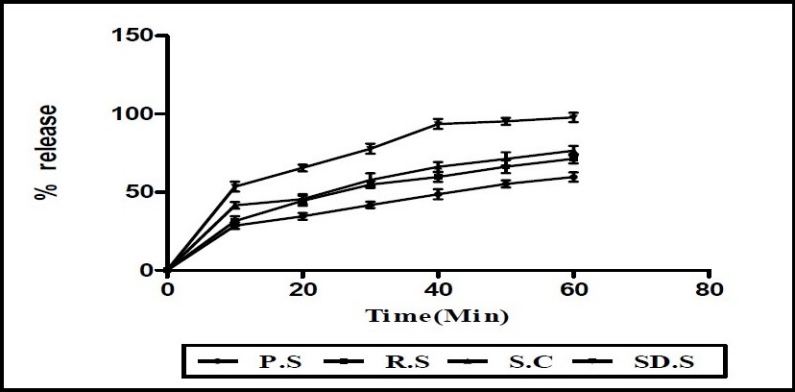
Dissolution of pure drug sample (P.S), recrystallized sample (R.S), spray chilled sample (S.C), spray dried sample (SD.S). n=3, each sample was determined in triplicate.

The dissolution profiles of piroxicam (Fig. 6) exhibited improved dissolution behavior for spray dried microparticles than spray chilled particles, recrystallized sample and pure sample. The dissolution of spray chilled particles was increased compared to recrystallized sample and pure sample but not much as compared to spray dried microparticles(21).

The results of the stability study of microparticles, stored at 20 °C and 45% relative humidity for 90 days, revealed that spray dried microparticles of piroxicam were stable after 90 days.

## DISCUSSION

The percentage yield of spray dried microparticles was found to be less compared to spray chilled particles. It could be increased by adding a solid substance or in large scale production, as in the present study the scale of preparation was small.

The DSC studies showed that piroxicam pure sample melted at 203.79 °C with enthalpy of 173.5 J/g, and melting point of recrystalized drug was at 203.46 with enthalpy of 169.32 J/g. The melting endotherm for spray dried microparticles of piroxicam was 199.43 °C with decreased enthalpy of 156.34 J/g, which indicated decreased crystallinity. The DSC thermogram of spray chilled piroxicam showed melting endotherm at the characteristic endothermic peak at 202.73 °C with enthalpy of 164.37 J/g indicating decreased crystallinity but not compared to spray dried microparticles, as spray dried microparticles showed more decreased crystallinity than spray chilled particles. These results showed the presence of amorphous form or decreased crystallinity of piroxicam in spray dried microparticles. The decreased in crystallinity was as follow: pure sample, recrystallized sample, spray chilled particles, spray dried microparticles.

Infrared spectra of commercial, recrystallized, spray chilled and spray dried microparticles of piroxicam showed characteristic peaks in the same position except in case of spray chilled particles. The Spectrum of spray chilled particle of piroxicam was slightly different from pure sample in the region of wavelength between 1630 and 1780 cm^-1^. This may suggest that the spray chilled particles of piroxicam, prepared by heating the drug sample, has a different crystalline form than its crystalline form in pure sample and in spray dried microparticles. It could be because of the degradation of the drug or variations in the resonance structure, rotation of a part of a molecule or certain bonds. Alteration could be due to minor distortion of bond angles.

X-Ray diffraction was used to analyze potential changes in the inner structure of piroxicam nanocrystal during the formulation. The extent of such changes depends on the chemical nature of the drug crystal ingredient. PXRD patterns of the unprocessed piroxicam, spray chilled particles and spray dried microparticles are shown in Fig. The characteristic peak of the piroxicam appeared in the 2θ range of 10-40^0^, indicating that the unprocessed piroxicam was a crystalline material. The XRD thermograph of pure piroxicam powder, recrystallized sample, chilled particles and spray dried microparticles showed that crystallanity of piroxicam was not affected significantly. The XRD spectra of recrytallized samples showed almost the same pattern as pure drug sample. There was not much difference, but in the XRD pattern of spray dried microparticles of piroxicam which showed absence, broadening and reduction of major piroxicam diffraction peak, indicating that mostly an amorphous from (disordered state) existed. The reduced crystallinity of piroxicam in the microparticles could explain the observed enhancement of solubility and dissolution of piroxicam microparticles.

In particle size determination, the result showed that spray dried microparticles were small and uniform in size compared to chilled particles and recrystallized particles, which contributed to the increase in the physicochemical properties of the prepared microparticles. The SEM result showed that spray dried microparticles were spherical and uniform in shape, which help in increasing the stability of the microparticles and protecting them from caking

Spray dried microparticles exhibited superior compressibility characteristics compared to pure sample and spray chilled particles. It could be due to the fact that during the process of compression, fresh surfaces are formed by fracturing crystals. The surface freshly prepared by fracture may enhance the plastic inter particle bonding, resulting in a lower compression force required for compressing the agglomerates under plastic deformation, compared to that of single crystals. Tensile strength of the piroxicam exhibited compressibility as follow: pure sample, chilled particles, spray dried microparticles.

The solubility studies revealed that spray drying technique has good ability to increase the solubility of poorly water soluble drugs, like piroxicam compare to chilling and recrystallization techniques. This above result showed higher solubility of spray dried microparticles and spray chilled particles than pure drug sample, which could be due to the increased wet-ability of microparticles. The higher solubility of spray dried microparticles compared to pure drug sample may be due to the presence of amorphous form of piroxicam in microparticles.

The dissolution profiles of piroxicam exhibited improved dissolution behavior for spray dried microparticles than spray chilled particles, recrystallized sample and pure sample. The reason for this faster dissolution could be linked to the better wet-ability of the microparticles. The amount of the drug dissolved in 60 min greatly varied for spray dried microparticles. The dissolution of spray chilled particles was increased compared to recrystallized sample and pure sample but not much as compared to spray dried microparticles. This could be due to the degradation of the drug by heating with less solubility characteristics compared to the spray dried microparticles. Therefore, based on these results together with the assumption of formation of melt-solidified bonds, the low dissolution from particles prepared by spray chilling technique could be explained.

The influence of microparticles on the physical stability of piroxicam was investigated. The drug fusion enthalpy of formed microparticles was decreased from 156.34 to 156.31 J/g after storage, with a subsequent decrease in piroxicam crystallinity, whereas in case of spray chilled particles, the drug fusion enthalpy was increased from 164.37 to 169.74 J/g after storage, which indicated an increase in crystallinity of piroxicam. The above result showed that spray dried microparticles of piroxicam were stable after 90 days at 20 °C and 45% relative humidity, but spray chilled particles were not stable during the storage period. stable during the storage period

## CONCLUSION

Spray dried microparticles and spray chilled particles of piroxicam were prepared to improve the dissolution rate. Microparticles exhibited decreased crystallinity and improved micromeritic properties. DSC and XRD studies showed that there was no change in the crystal structure of piroxicam during the spray drying process i.e., polymorphism has not occurred. The dissolution of the spray dried microparticles was improved compared with spray chilled particles, recrystallized and pure samples. Spray chilling of piroxicam reduced the drug release compared to the spray dried microparticles. This could be due to the degradation of drug by heating or variations in the resonance structure, rotation of a part of a molecule or certain bonds. Alteration could be due to minor distortion of bond angles. Hence, spray chilling is not a suitable technique to improve dissolution of piroxicam, compare to spray drying technique. The spray dried microparticles, but not spray chilled particles, were stable after 90 days of storage.

Hence, this spray drying technique can be used for formulation of tablets of piroxicam by direct compression with directly compressible tablet excipients, and without further processing like, mixing and granulation.
